# Exploration of the cysteine reactivity of human inducible Hsp70 and cognate Hsc70

**DOI:** 10.1016/j.jbc.2022.102723

**Published:** 2022-11-19

**Authors:** Zhouping Hong, Weibin Gong, Jie Yang, Sainan Li, Zhenyan Liu, Sarah Perrett, Hong Zhang

**Affiliations:** 1 National Laboratory of Biomacromolecules, CAS Center for Excellence in Biomacromolecules, Institute of Biophysics, Chinese Academy of Sciences, Beijing, China; 2 University of the Chinese Academy of Sciences, Beijing, China

**Keywords:** cysteine reactivity, Hsp70, glutathionylation, disulfide bond formation, oxidative stress, CD, circular dichroism, DTNB, 5,5′-dithiobis-(2-nitrobenzoic acid), FP, fluorescence polarization, MB, methylene blue, NBD, nucleotide-binding domain, PES, 2-phenylethynesulfonamide, PTM, posttranslational modification, RT, room temperature, SBD, substrate-binding domain, SEC, size-exclusion chromatography

## Abstract

Hsp70s are multifunctional proteins and serve as the central hub of the protein quality control network. Hsp70s are also related to a number of diseases and have been established as drug targets. Human HspA1A (hHsp70) and HspA8 (hHsc70) are the major cytosolic Hsp70s, and they have both overlapping and distinct functions. hHsp70 contains five cysteine residues, and hHsc70 contains four cysteine residues. Previous studies have shown these cysteine residues can undergo different cysteine modifications such as oxidation or reaction with electrophiles to regulate their function, and hHsp70 and hHsc70 have different cysteine reactivity. To address the mechanism of the differences in cysteine reactivity between hHsp70 and hHsc70, we studied the factors that determine this reactivity by Ellman assay for the quantification of accessible free thiols and NMR analysis for the assessment of structural dynamics. We found the lower cysteine reactivity of hHsc70 is probably due to its lower structural dynamics and the stronger inhibition effect of interaction between the α-helical lid subdomain of the substrate-binding domain (SBDα) and the β-sheet substrate-binding subdomain (SBDβ) on cysteine reactivity of hHsc70. We determined that Gly557 in hHsp70 contributes significantly to the higher structural dynamics and cysteine reactivity of hHsp70 SBDα. Exploring the cysteine reactivity of hHsp70 and hHsc70 facilitates an understanding of the effects of redox reactions and electrophiles on their chaperone activity and regulation mechanisms, and how these differences allow them to undertake distinct cellular roles.

Hsp70s are highly conserved and found in all living things apart from some archaea (1, 2). Hsp70s are important for life, and they are multifunctional proteins. The most dominant functions of Hsp70s are assisting *de novo* protein folding, antagonizing stress, and buffering exogenous and endogenous stimulation (3). Hsp70s are the central hub of the protein quality control network, and they can interact with numerous clients to chaperone and facilitate their proper activity, contributing to proteostasis (4). Hsp70s are also involved in a number of diseases and are established drug targets, including for cancer, neurodegenerative diseases, and infectious diseases, due to the participation of Hsp70s in maintaining the high tolerance of cancer cells, preventing and removing aggregates and modulating the immune response (5, 6).

Hsp70s are often conserved in sequence and structure with evolution (7). The canonical Hsp70 structure contains an N-terminal nucleotide-binding domain (NBD) and a C-terminal substrate-binding domain (SBD) joined by a flexible linker (Fig. 1*A*). Hsp70 is a typical allosteric protein and its conformation is affected by nucleotide and substrate binding (8). In the ATP-bound state, the two domains of Hsp70 tend to dock together, while in the ADP-bound state, the two domains generally remain independent (9, 10). When the SBD is docked with the NBD, its α-helical lid subdomain (SBDα) and β-sheet substrate-binding subdomain (SBDβ) come apart and the interaction between the NBD and SBDβ results in low substrate affinity and low ATPase activity (9). However, substrate binding to ATP-bound Hsp70 weakens interaction between the NBD and SBDβ, which leads to undocking of the NBD and SBD and promotes ATP hydrolysis, and the resulting ADP-bound Hsp70 has high substrate affinity (11). ATP-ADP exchange in the NBD helps Hsp70 to recover to the ATP-bound state, releasing the bound substrate (12). The above processes form the functional cycle of Hsp70, and different cochaperone Hsp40s and nucleotide exchange factors finely tune this cycle by interacting with Hsp70 (3).

**Figure 1:**
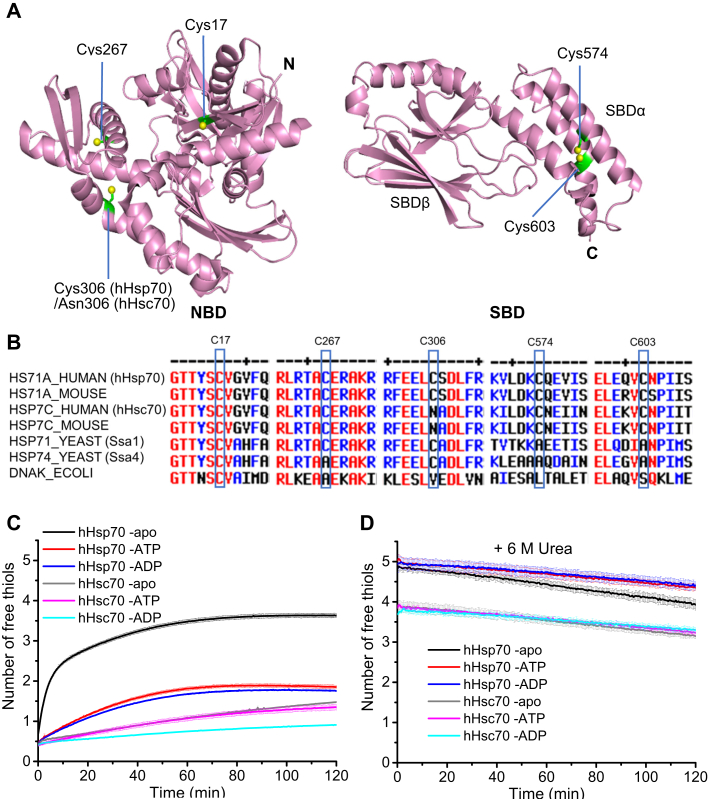
**Comparison of overall cysteine reactivity of hHsp70 and hHsc70.***A*, the cysteine residues of hHsp70 and hHsc70 are labeled in the crystal structures of the hHsp70 nucleotide-binding domain (NBD) in the ADP-bound state (PDB code 3AY9) and the substrate-binding domain (SBD, PDB code 4PO2). Cys17, Cys267, Cys574, and Cys603 exist in both hHsp70 and hHsc70, while Cys306 only exists in hHsp70. *B*, sequence alignments of hHsp70, hHsc70, Hsp70, and Hsc70 from mouse, Ssa1 and Ssa4 from *Saccharomyces cerevisiae*, and DnaK from *Escherichia coli* showing regions across the cysteine residues. *C* and *D*, the time course of cysteine reactivity of hHsp70 and hHsc70 in the absence and presence of 1 mM ADP or ATP in the native state (*C*) or in 6 M urea (*D*) were monitored in a plate reader and compared. The number of free thiols was calculated according to a standard curve by Ellman assay. The data shown are the mean of three independent measurements and the error bars represent the SD.

HspA1A and HspA8 are the best studied human cytosolic Hsp70s. Human HspA1A is stress inducible, and we term it hHsp70 in this article. Human HspA8, also called 71 kDa heat shock cognate (Hsc70), is the housekeeping Hsp70, and we term it hHsc70. Although these two Hsp70s share 86% sequence identity and have overlapping functions, they also have specific functions and some differences in their properties (7). hHsp70 tends to respond to stress, while hHsc70 contributes to fundamental cellular activities such as clathrin-mediated endocytosis and chaperone-mediated autophagy (7). Proteome-wide analysis has shown that under normal growth conditions, clients of hHsp70 and hHsc70 are largely nonoverlapping, although both of these Hsp70s prefer to associate with newly synthesized polypeptides (13).

Posttranslational modifications (PTMs) of chaperones, known as the chaperone code, finely regulate chaperone activity to integrate molecular chaperones into the cellular signaling network, since PTMs are often involved in signal transduction pathways (14, 15). For example, cysteine residues often undergo different oxidative cysteine modifications to transfer redox information (16, 17). Cysteine is unique in its nucleophilicity and can undergo oxidative modifications upon redox and covalent modifications with electrophiles including certain drugs (18). Most Hsp70s contain at least one cysteine residue and the number of Cys residues in Hsp70s increases with evolution. Proteomics have identified an array of cysteine modifications of Hsp70s (19, 20, 21, 22, 23). Yeast ER Hsp70 BiP undergoes S-sulfenation (-SOH) and glutathionylation to enhance holdase activity of BiP to prevent protein aggregation during oxidative stress (19, 24). Our previous studies have shown that glutathionylation of the *Escherichia coli* Hsp70 homolog DnaK can link oxidative stress and the heat shock response by reversibly regulating chaperone activity and interaction between DnaK and its cochaperones (25). We also found that glutathionylation of Cys residues in the SBDα of hHsp70 reversibly blocks its substrate-binding cleft and turns off the chaperone activity, indicating that chaperone activity of hHsp70 can be regulated by redox (21). Further, Cys residues in the SBDα of hHsp70 can be covalently modified by the electrophile PES (2-phenylethynesulfonamide or pifithrin-μ) to turn off the chaperone activity of hHsp70 by a similar mechanism as glutathionylation (26).

It is important to explore the cysteine reactivity of a protein to know the propensity of individual cysteine residues to undergo cysteine modification and to evaluate how the protein is affected by redox and electrophiles (27). Cysteine reactivity is determined by the pKa of the cysteine thiol group as well as the protein microenvironment around the cysteine thiol (*i.e.*, whether it is in a buried/hydrophobic environment, or an exposed/hydrophilic one, or somewhere in between) (28). In this study, we compared the cysteine reactivity of individual cysteine residues in hHsp70 and hHsc70, explored the factors affecting cysteine reactivity, and further compared glutathionylation and intramolecular disulfide bond formation in hHsp70 and hHsc70 to help understand the mechanistic basis of the functional differences between hHsp70 and hHsc70.

## Results

### hHsp70 and hHsc70 have different cysteine reactivity

**Figure 2:**
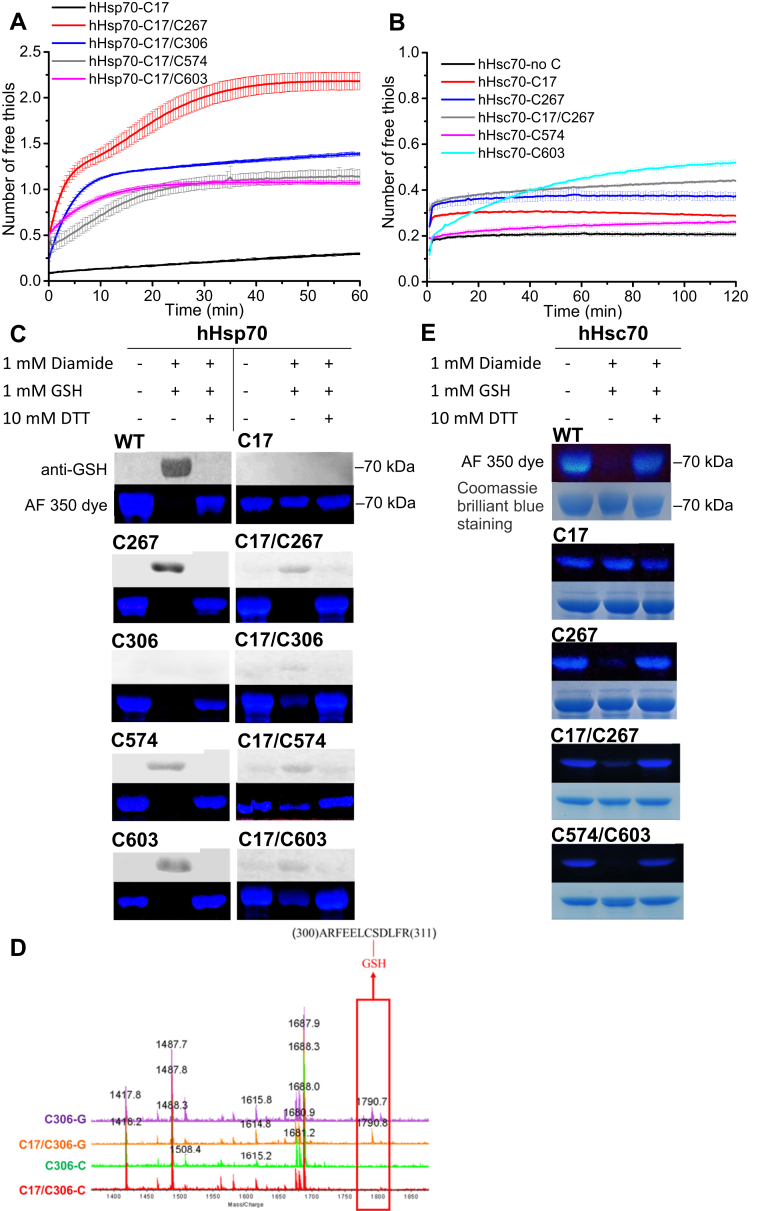
**Comparison of cysteine reactivity and glutathionylation of individual Cys residues in hHsp70 and hHsc70.***A* and *B*, the time course of cysteine reactivity of hHsp70 mutants containing one or two Cys residues (*A*) and hHsc70 mutants without Cys or containing one Cys (*B*) were monitored in a plate reader and the number of free thiols was calculated. The data shown are the mean of three independent measurements and the error bars represent the SD. *C*, Western blot detection and Alexa Fluor 350 dye (AF 350 dye) staining distinguish glutathionylation and nonglutathionylation of WT hHsp70 and its mutants containing one or two Cys; 1:500 anti-GSH was used in the Western blot detection. *D*, MALDI-TOF detection of glutathionylation of Cys306 in hHsp70. Untreated control (-C) and glutathionylated (-G) hHsp70 mutants which contain Cys306 or both Cys17 and Cys306 but not other cysteine residues were checked. *E*, AF 350 dye staining distinguishes glutathionylation and nonglutathionylation of WT hHsc70 and hHsc70 mutants containing one or two Cys.

hHsp70 has five Cys residues (Cys17, Cys267, and Cys306 in the NBD and Cys574 and Cys603 in the SBDα), while hHsc70 has four Cys residues (Cys17, Cys267 in the NBD and Cys574 and Cys603 in the SBDα) (Fig. 1*A*). Cys306, which exists in hHsp70 but not in hHsc70, was found to distinguish redox sensitivity between hHsp70 and hHsc70 (29). Our previous results indicate that hHsp70 has higher cysteine reactivity than hHsc70 (21, 26). The sequence alignment across the cysteine residues shows that the sequence conservation is very high among mammalian cytosolic stress–induced Hsp70 and Hsc70 but is not as high between mammalian Hsp70 and either yeast or *E. coli* Hsp70 (Fig. 1*B*). Here, we compared the cysteine reactivity of native hHsp70 and hHsc70 in the apo, ATP-bound, and ADP-bound states and found hHsp70 has obviously higher cysteine reactivity than hHsc70 especially in the apo state (Fig. 1*C*). However, under denatured conditions (in the presence of 6 M urea), the detected number of free thiols per hHsp70 or hHsc70 molecule in the apo, ATP-bound, or ADP-bound state almost equals the actual number of Cys residues in the Hsp70 molecule (Fig. 1*D*). These results suggest that the cysteine reactivity of each Cys in hHsp70 and hHsc70 is affected by the native conformation. We then carefully explored the cysteine reactivity of each Cys in hHsp70 and hHsc70. Using hHsp70 and hHsc70 mutants containing only Cys17 but not the other Cys residues, we found the single Cys17 has very low cysteine reactivity in reaction with 5,5′-dithiobis-(2-nitrobenzoic acid) (DTNB) and could not undergo glutathionylation upon diamide and GSH treatment (Fig. 2, *A*–*C* and *E*). Using hHsp70 and hHsc70 mutants containing one of the other Cys residues, with or without Cys17, we found that each of the other individual Cys residues besides Cys17 showed cysteine reactivity in DTNB assays and/or glutathionylation reaction upon diamide and GSH treatment (Fig. 2). Glutathionylation of Cys306 in hHsp70 was not sensitive to Western blot detection with anti-GSH antibody although reaction of free thiols with Alexa Fluor 350 (AF 350) dye showed there was no free thiol in hHsp70-C306 after diamide and GSH treatment (Fig. 2*C*). Therefore, we used MALDI-TOF MS to detect glutathionylation of Cys306 in hHsp70 and found glutathionylated hHsp70-C306 and hHsp70-C17/C306 contained a clear additional peak corresponding to the peptide containing glutathionylated Cys306, compared with the untreated hHsp70-C306 and hHsp70-C17/C306 (Fig. 2*D*). This suggests that the conserved Cys17 present in both hHsp70 and hHsc70 shows no independent cysteine reactivity if the other Cys residues are mutated, while the other Cys residues have independent reactivity. In the DTNB assay, the reaction rate (the slope of the reaction curve) and the detected free thiol number per molecule can reflect cysteine reactivity, and we found that Cys267, Cys574, and Cys603 have much higher cysteine reactivity in hHsp70 than in hHsc70 (Fig. 2, *A* and *B*). This suggests that Cys residues are more readily exposed in hHsp70 than in hHsc70, implying that hHsp70 has a higher degree of flexibility in terms of structural dynamics than hHsc70.

Using hHsp70 mutants containing Cys17 and one of the other Cys, we found that after glutathionylation reaction upon diamide and GSH treatment, hHsp70-C17/C306, hHsp70-C17/C574, and hHsp70-Cys17/C603 still contained free thiols which could be labeled by AF 350 fluorescent dye, while hHsp70-Cys17/C267 did not contain any free thiols (Fig. 2*C*). This indicates that in hHsp70, modification of Cys17 can only occur as a secondary step subsequent to modification of Cys267. Consistent with this, DTNB assay of hHsp70-C17/C267 showed cysteine reactivity equivalent to about two free thiols and hHsp70-C17/C306, hHsp70-C17/C574, and hHsp70-C17/C603 each showed cysteine reactivity equivalent to only around one free thiol (Fig. 2*A*). In hHsc70, we noticed that in DTNB assays, hHsc70-C17/C267 has higher cysteine reactivity than hHsc70-C267 (Fig. 2*B*). However, after diamide and GSH treatment, hHsc70-C17/C267 still has free thiols, and WT hHsc70 in the apo state has no free thiols (Fig. 2*E*). This suggests that modification of Cys17 is facilitated by modification of the other three Cys residues. In contrast, the single conserved Cys15 in *E. coli* DnaK (equivalent to Cys17 in human Hsp70) has some cysteine reactivity in the absence of nucleotide (25), suggesting a change during the course of evolution in the function of the highly conserved N-terminal Cys residue.

Glutathionylation of Cys574 and Cys603 in the SBDα causes fully reversible structural and functional changes in hHsp70 (21). Here, we found that glutathionylation of Cys residues in the NBD of hHsp70 (Cys17, Cys267, and Cys306) causes a decrease in nucleotide-binding ability, ATPase activity, and substrate-binding ability (Figs. S1, S2 and Table S1). For Cys17 and Cys267, the effect of glutathionylation is partially reversible and accompanies aggregation of hHsp70, while for Cys306, the effect is slight and fully reversible without inducing aggregation (Figs. S1, S2 and Table S1). In WT hHsp70, glutathionylation of all five Cys residues causes partially reversible changes (Figs. S1, S2 and Table S1).

Similar to hHsp70, glutathionylation of Cys17 and Cys267 caused partially reversible aggregation of hHsc70, and glutathionylation of Cys574 and Cys603 caused reversible structural changes in hHsc70 (Fig. S3). It is interesting to note that glutathionylation at Cys267 caused irreversible aggregation of hHsc70, whereas glutathionylation of all four Cys residues did not cause aggregation of hHsc70 (Fig. S3). Consistent with this, PTMs of Hsp70 have been assumed to play roles in the oligomerization of Hsp70 proteins (30). PTMs including glutathionylation often alter the structure of Hsp70, possibly resulting in exposure of some hydrophobic sites and further oligomerization. Even without PTMs such as glutathionylation, Hsp70 has a tendency to form oligomers and large aggregates. We found that DTNB assays could not be applied to the large aggregates of hHsp70 and hHsc70 as this interfered with absorbance measurements, whereas the smaller oligomeric and monomeric forms obtained in the last SEC purification step of hHsp70 or hHsc70 showed similar levels of activity in DTNB assays.

### hHsp70 and hHsc70 show different effects of nucleotide and adjacent domains on cysteine reactivity

**Figure 3:**
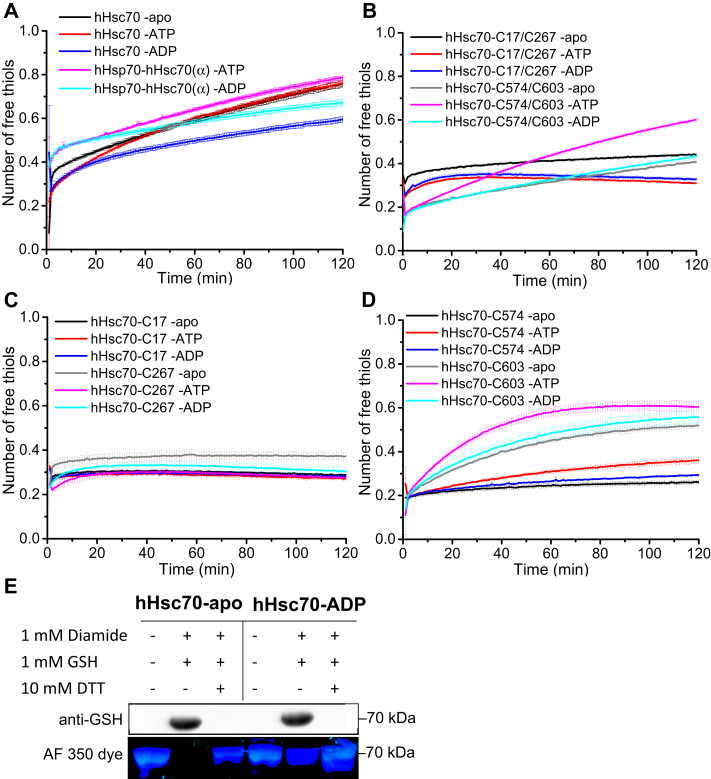
**The effect of nucleotide on cysteine reactivity of hHsc70.***A*–*D*, the time course of cysteine reactivity of WT hHsc70 or its mutants in the absence and presence of 1 mM ADP or ATP was monitored in a plate reader and the number of free thiols was calculated. The data shown are the mean of three independent measurements and the error bars represent the SD. WT hHsc70 and chimeric protein containing the NBD and SBDβ of hHsp70 and the SBDα of hHsc70 (hHsp70-hHsc70(α)) were compared in (*A*), and hHsc70 mutants containing two Cys in the NBD or SBD (*B*) or a single Cys (*C* and *D*) were used to check the effect of nucleotide on cysteine reactivity in the NBD and SBD of hHsc70 individually. *E*, Western blot detection and Alexa Fluor 350 dye (AF 350 dye) staining distinguish glutathionylation and nonglutathionylation of WT hHsc70 in the absence of nucleotide and in the presence of 1 mM ADP; 1:500 anti-GSH was used in the Western blot detection. NBD, nucleotide-binding domain; SBD, substrate-binding domain.

The cysteine reactivity of hHsp70 is affected by nucleotide and influenced by the presence of the adjacent domains (21, 26), so we focused here on the comparison between hHsp70 and hHsc70. We noticed that ADP and ATP have different effects on the cysteine reactivity of hHsc70, while ADP and ATP have the same effect on cysteine reactivity of hHsp70 as detected by Ellman assays (Figs. 1*C* and 3*A*). In DTNB assays, the presence of ADP and ATP had no effect on the cysteine reactivity of hHsc70-C17 and decreased the cysteine reactivity of hHsc70-C17/C267 and hHsc70-C267 (Fig. 3, *B* and *C*). For hHsc70-C574/C603, hHsc70-C574, and hHsc70-C603, ADP had no effect on the cysteine reactivity and ATP increased the cysteine reactivity (Fig. 3, *B* and *D*). When we checked glutathionylation by Western blot and AF 350 dye, we found that in the absence of nucleotide, all of the Cys residues in WT hHsc70 underwent glutathionylation, while in the presence of ADP, only a proportion of Cys residues underwent glutathionylation (Fig. 3*E*). When we checked glutathionylation by SEC, we found the presence of ADP inhibited the conformational changes and glutathionylation of hHsc70-C267 but not WT hHsc70, hHsc70-C574, and hHsc70-C603 (Fig. S3). Thus, ADP inhibited the cysteine reactivity and glutathionylation of the Cys residues in the NBD but not the SBD of hHsc70, similar to hHsp70. ATP also inhibited cysteine reactivity in the NBD of hHsc70 but promoted cysteine reactivity in the SBD of hHsc70. We constructed a chimera composed of the SBDα of hHsc70 and the NBD and SBDβ of hHsp70, termed hHsp70-hHsc70(α). We found that ADP and ATP also have different effects on cysteine reactivity of hHsp70-hHsc70(α), similar to hHsc70 (Fig. 3*A*). Hsp70 has two major conformations and the interaction between the SBDα and SBDβ is stronger in the ADP-bound state than in the ATP-bound state (9, 10, 11). This suggests that the interaction between the SBDα and SBDβ has a more obvious inhibition effect on cysteine reactivity of the hHsc70 SBDα than on the hHsp70 SBDα, and the interaction between the SBDα of hHsc70 and the SBDβ from either hHsc70 or hHsp70 can inhibit cysteine reactivity of the hHsc70 SBDα.

**Figure 4:**
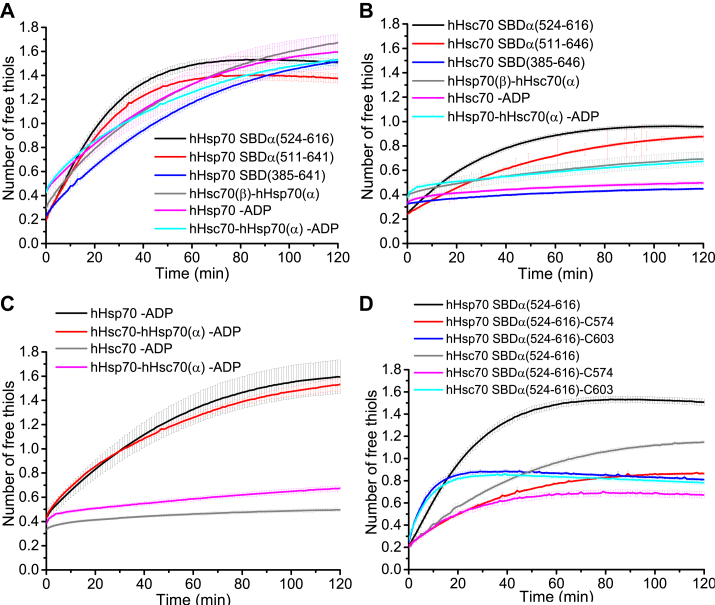
**The effect of adjacent domains on cysteine reactivity in SBDα of hHsp70 and hHsc70.***A*, the time course of cysteine reactivity in the SBDα of hHsp70 was measured for WT hHsp70 and chimeric protein containing the NBD and SBDβ of hHsc70 and SBDα of hHsp70 (hHsc70-hHsp70(α)) in the presence of 1 mM ADP and in different hHsp70 truncation mutants or chimera containing the SBDα of hHsp70, as indicated. *B*, the time course of cysteine reactivity of the SBDα of hHsc70 was measured for WT hHsc70 and chimeric protein containing the NBD and SBDβ of hHsp70 and SBDα of hHsc70 (hHsp70-hHsc70(α)) in the presence of 1 mM ADP and in different hHsc70 truncation mutants or chimeras containing the SBDα of hHsc70, as indicated. *C*, the time course of cysteine reactivity of the SBDα of hHsp70 and hHsc70 were compared for WT hHsp70, hHsc70-hHsp70(α), WT hHsc70, and hHsp70-hHsc70(α) in the presence of 1 mM ADP. *D*, the time course of cysteine reactivity of the SBDα from hHsp70 and hHsc70 were compared for hHsp70 SBDα(524-616), hHsc70 SBDα(524-616), and their mutants containing single Cys. The data shown are the mean of three independent measurements and the error bars represent the SD. NBD, nucleotide-binding domain.

To further study the effect of domain interaction on cysteine reactivity of hHsp70 and hHsc70, we measured and compared the cysteine reactivity of the SBDα in different constructs containing the SBDα from hHsp70 or hHsc70. SBDα(524-616) consisting of α-helix B, C, and D, and including Cys574 and Cys603, is the major part of the SBDα. For hHsp70, we found that SBDα(524-641) shows similar cysteine reactivity to SBDα(524-616), and adding the SBDβ from hHsp70 or hHsc70 slowed down the reaction rate in the DTNB assay (Fig. 4*A*). If the NBD from hHsp70 or hHsc70 was further added, and in the presence of ADP, the cysteine reactivity of the NBD was inhibited, and the observed level of cysteine reactivity was similar to the isolated SBD (Fig. 4*A*). For hHsc70, SBDα(524-641) had a slower reaction rate towards DTNB than SBDα(524-616), and adding the SBDβ from hHsc70 had a stronger inhibition effect on the cysteine reactivity, compared with adding the SBDβ from hHsp70 (Fig. 4*B*). Similar to the situation for hHsp70, adding the NBD from hHsc70 or hHsp70 did not have any significant effect on cysteine reactivity (Fig. 4*B*). In summary, it was found that adding adjacent domains to SBDα(524-616) weakened the cysteine reactivity of the SBDα in both hHsp70 and hHsc70, and the inhibition effect from adjacent domains was much more obvious in hHsc70 than in hHsp70 (Fig. 4, *A–C*). These results are consistent with the different effects of ADP and ATP on the cysteine reactivity of the SBDα from hHsp70 and hHsc70. Therefore, domain interaction has a more significant effect on the cysteine reactivity of the SBDα from hHsc70 than from hHsp70.

We noticed that besides the stronger inhibition effect from domain interactions, cysteine reactivity of hHsc70 SBDα(524-616) was still obviously lower than that of hHsp70 SBDα(524-616) (Fig. 4*D*). We then compared the cysteine reactivity of hHsp70 and hHsc70 SBDα(524-616) mutants containing only Cys574 or Cys603 and found cysteine reactivity of Cys603 was almost the same in the SBDα(524-616) of hHsp70 and hHsc70, while the cysteine reactivity of Cys574 in the SBDα(524-616) was lower for hHsc70 than for hHsp70 (Fig. 4*D*). Therefore, the different cysteine reactivity of hHsc70 SBDα and hHsp70 SBDα seems to come from different cysteine reactivity of Cys574, but not Cys603. In hHsp70, Cys603 is more reactive than Cys574, and Cys574 has a greater contribution to structural stability of SBDα (21). In this study, we found this is also the case for hHsc70.

### The different structural dynamics of hHsp70 SBDα and hHsc70 SBDα affects their cysteine reactivity

**Figure 5:**
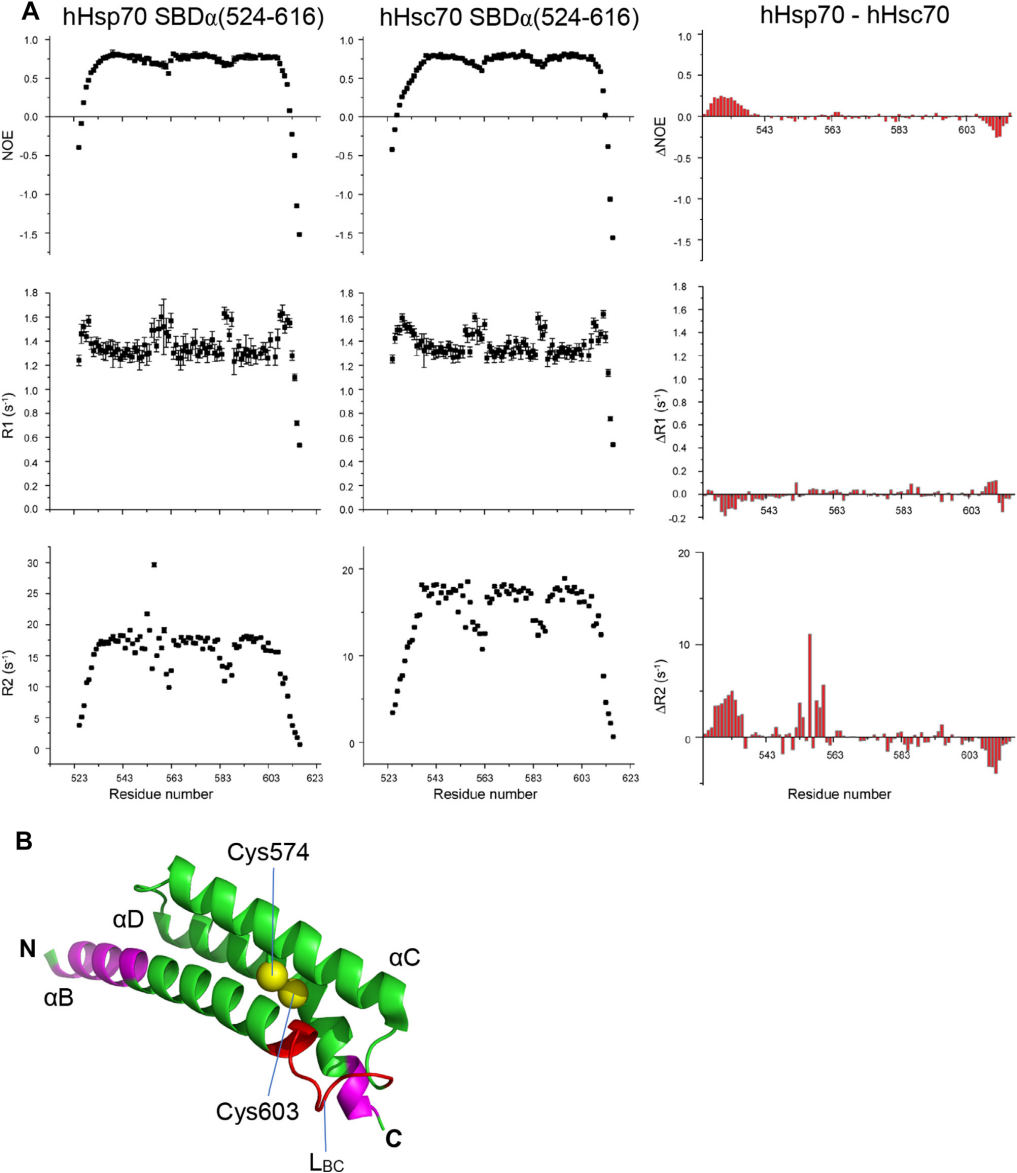
**The regions affecting conformational dynamics of the SBDα of hHsp70 and hHsc70.***A*, steady-state dynamics of hHsp70 SBDα(524-616) and hHsc70 SBDα(524-616) were compared using NMR spectroscopy. *B*, the structure of hHsp70 SBDα (PDB code 4PO2) with labeling (in *red* or in *magenta*) of the regions showing different dynamics in (*A*). Sulfur atoms of Cys574 and Cys603 are shown as *spheres*. Residues near Cys574 and Cys603 are colored in *red*, while residues distant from Cys574 and Cys603 are in *magenta*.

**Figure 6:**
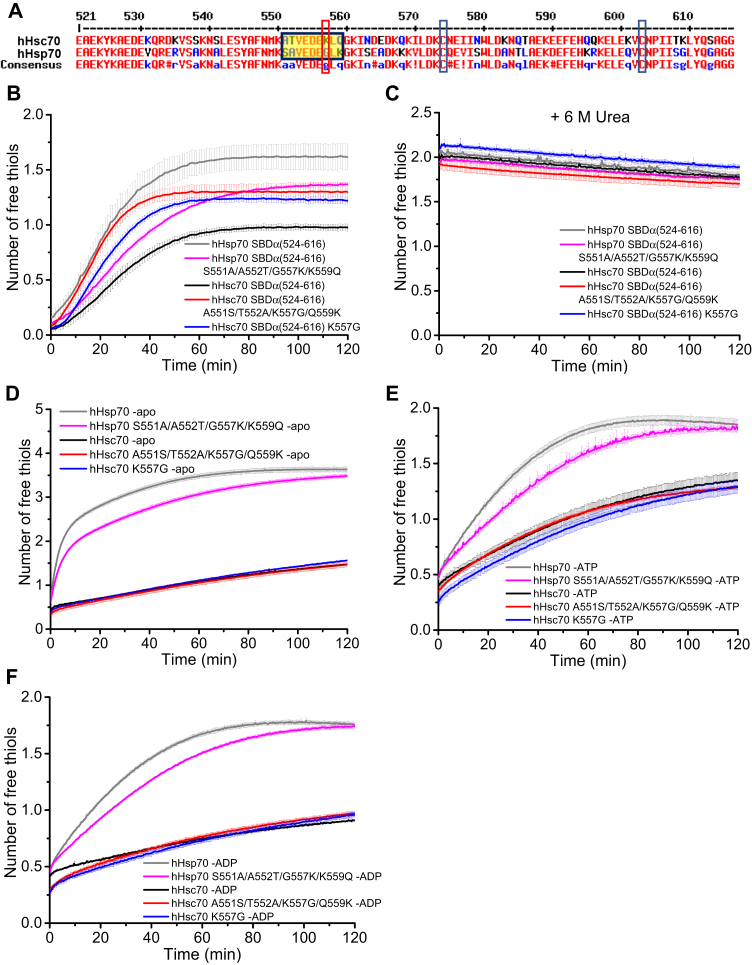
**The key residues affecting cysteine reactivity in the SBDα of hHsp70 and hHsc70.***A*, sequence alignment of the SBDα of hHsp70 and hHsc70. Cys574 and Cys603 are indicated by *dark blue boxes*. The key residues in L_BC_ as indicated in Figure 5*B* affecting conformational dynamics of SBDα are highlighted in *yellow* and indicated by a *red box*. *B* and *C*, the time course of cysteine reactivity of hHsp70 SBDα(524-616), hHsc70 SBDα(524-616), and their mutants with exchange of the key residues L_BC_ of SBDα were compared in the native state (*B*) or in 6 M urea (*C*). *D*–*F*, the time course of cysteine reactivity of hHsp70, hHsc70, and their mutants with exchange of the key residues in L_BC_ of SBDα were compared in the absence (*D*) and presence of 1 mM ATP (*E*) or ADP (*F*). The data shown are the mean of three independent measurements and the error bars represent the SD.

To further explore the mechanisms for the different cysteine reactivity of the SBDα of hHsp70 and hHsc70, we measured NMR steady-state dynamics of SBDα(524-616) from hHsp70 and hHsc70, including HN-NOE, R1, and R2 (Fig. 5*A*). Three regions in SBDα(524-616) were found to be different between hHsp70 and hHsc70 (Figs. 5, *A*, *B* and 6*A*). The first region is the N-terminal helix of αB (residues 524-534) (Figs. 5, *A*, *B* and 6*A*). In this region, hHsp70 had higher NOEs and R2 values than hHsc70 (Fig. 5*A*), suggesting this region of hHsp70 has lower flexibility and a larger degree of conformational exchange. The main sequence difference in this region is residue 531 which is Val in hHsp70 and Lys in hHsc70 (Fig. 6*A*). The second region is the loop L_BC_ between αB and αC (Figs 5, *A*, *B* and 6*A*). hHsp70 L_BC_ shows significantly higher R2 values than hHsc70 L_BC_ (Fig. 5, *A* and *B*), suggesting significant conformational exchange of hHsp70 L_BC_ compared to hHsc70 L_BC_. The sequence of this region (residues 551-559) is SAVEDEGLK in hHsp70 and is ATVEDEKLQ in hHsc70 (Fig. 6*A*), of which Gly557 in hHsp70 and Lys557 in hHsc70 is possibly the key difference between the two sequences (Fig. 6*A*). The third region lies in the C terminal region, which showed a slightly higher R2 value for hHsc70 (Fig. 5, *A* and *B*).

Looking at the position of the abovementioned regions in the SBDα structure, the regions at the N-terminus and the C-terminus are far from Cys574 in the hydrophobic core, while the loop L_BC_ is close to Cys574 (Fig. 5*B*). Thus, we suspected that the difference in cysteine reactivity of the SBDα in hHsp70 and hHsc70 is likely to be caused by the residue difference in the loop L_BC_, since L_BC_ is near to both Cys574 and Cys603 (Fig. 5*B*). The loop L_BC_ of hHsp70 contains one Gly residue (Gly557) (Fig. 6*A*), which may allow the loop to easily undergo conformational exchange, increasing the accessibility of Cys574/Cys603 in the hydrophobic core of the α-helix bundle. In comparison, the corresponding residue 557 in hHsc70 is a lysine, not as flexible as a glycine residue. This might explain the higher cysteine reactivity of the SBDα in hHsp70 compared to hHsc70.

To verify whether the difference in residues 551-559 between hHsp70 and hHsc70 leads to the different cysteine reactivities of the SBDα, we swapped residues 551-559 between hHsp70 SBDα(524-616) and hHsc70 SBDα(524-616) and also constructed the hHsc70 SBDα(524-616) point mutant, K557G, and then compared the cysteine reactivities with the original hHsp70 SBDα(524-616) and hHsc70 SBDα(524-616) constructs (Fig. 6*B*). We found that for hHsc70 SBDα(524-616), both the quadruple mutation A551S/T552A/K557G/Q559K and the single mutation K557G increased the reaction rate in the DTNB assay indicating increased cysteine reactivity, while for hHsp70 SBDα(524-616), the inverse S551A/A552T/G557K/K559Q quadruple mutation decreased the reaction rate in the DTNB assay indicating decreased cysteine reactivity (Fig. 6*B*). We also compared the cysteine reactivity of these mutants, and of hHsp70 SBDα(524-616) and hHsc70 under denaturing condition (6 M urea), and found that the five proteins had similar cysteine reactivity after unfolding (Fig. 6*C*). Therefore, the residues 551-559, especially Gly557, which has a smaller sidechain group than Lys557, enable the loop region to be more dynamic in the native structure, leading to higher cysteine reactivity of the SBDα in hHsp70 than in hHsc70. To determine whether the difference in residues 551-559 between hHsp70 and hHsc70 also affects the cysteine reactivity of Cys574 and Cys603 in full-length hHsp70 and hHsc70, we introduced the same mutations in full-length hHsp70 and hHsc70 and then compared the cysteine reactivity of the new mutants with WT hHsp70 or WT hHsc70 in the absence and presence of ADP or ATP. We found for hHsp70, the S551A/A552T/G557K/K559Q quadruple mutation decreased the reaction rate in the DTNB assay, with a similar decrease in cysteine reactivity in full-length hHsp70 as in hHsp70 SBDα(524-616), while for full-length hHsc70, both the quadruple mutation A551S/T552A/K557G/Q559K and the single mutation K557G did not show any obvious effect on the cysteine reactivity in the absence or presence of nucleotide (Fig. 6, *D*–*F*). This indicates that the S551A/A552T/G557K/K559Q quadruple mutation restricts the conformational dynamics of SBDα in full-length hHsp70, while the inverse mutations cannot increase the conformational dynamics of SBDα in full-length hHsc70. This result also suggests that domain interactions have a more significant inhibition effect on cysteine reactivity and conformational dynamics of the SBDα in hHsc70 than in hHsp70, consistent with the results described above.

### Cys574 and Cys603 in both hHsp70 and hHsc70 can undergo multiple Cys modifications

**Figure 7:**
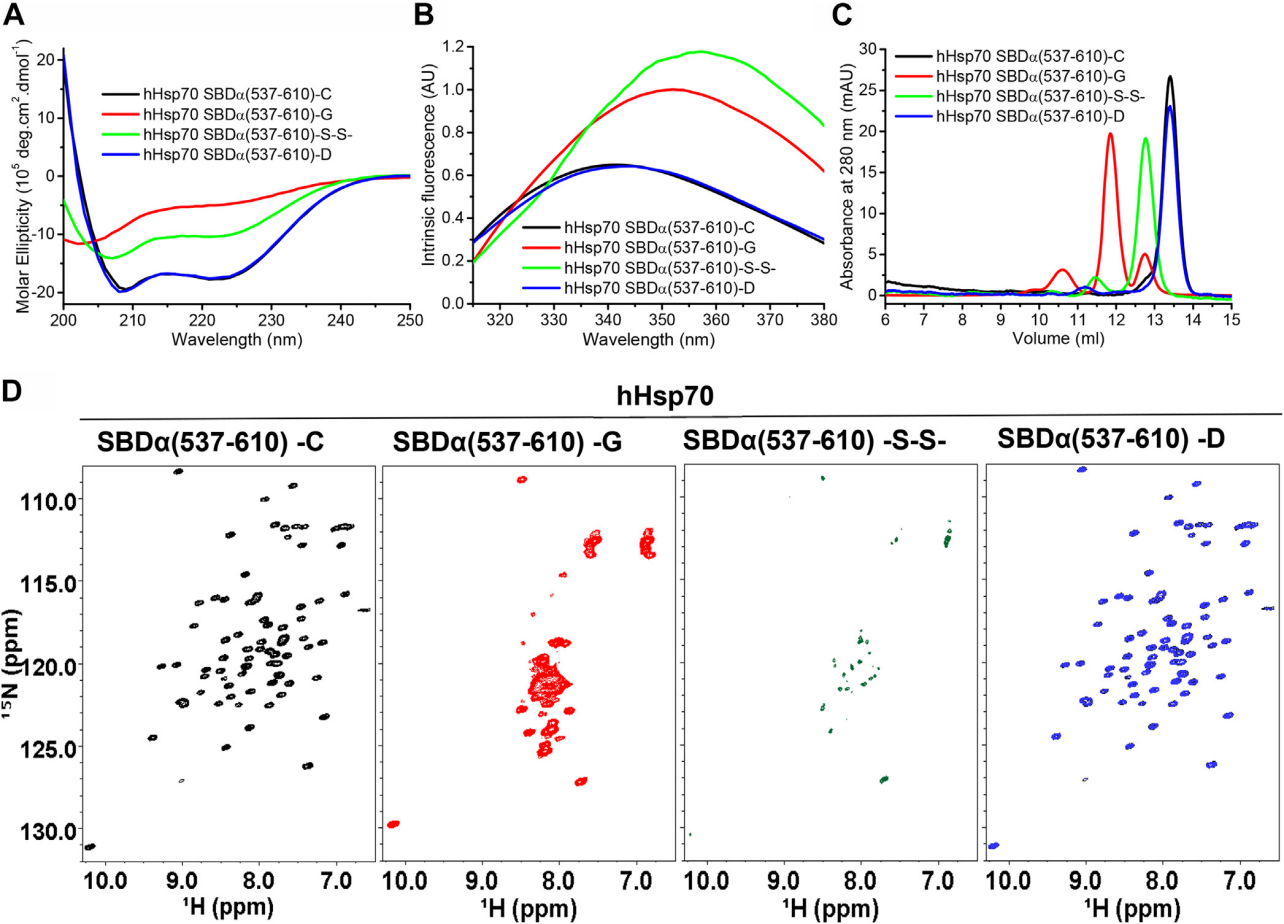
**Comparison of the effects of glutathionylation and intramolecular disulfide bond formation involving Cys574 and Cys603 on the structure of hHsp70 SBDα(537-610).***A*–*C*, the conformation and secondary structure of untreated control (-C, *black*), glutathionylated (-G, *red*), disulfide-bonded (-S-S-, *green*), and DTT-reduced (-D, *blue*) SBDα(537-610) were compared by far-UV CD (*A*), intrinsic tryptophan fluorescence (after excitation at 295 nm) (*B*), and SEC (C). Proteins of 20 μM were loaded onto a 24-ml Superdex 75 10/300 Gl column. The elution profiles were calibrated using blue dextran (2000 kDa, 7.50 ml), beta-amylase (200 kDa, 8.35 ml), alcohol dehydrogenase (150 kDa, 8.80 ml), bovine serum albumin (66 kDa, 9.56 ml), ovalbumin (45 kDa, 10.48 ml), carbonic anhydrase (29 kDa, 11.79 ml), PMSF-treated trypsinogen (24 kDa, 12.60 ml), and cytochrome c (12.4 kDa, 13.54 ml). The peak position of monomeric hHsp70 SBDα(537-610) (10 kDa) was 13.41 ml, and the peaks at 12.77 ml and 11.84 ml correspond to monomeric hHsp70 SBDα(537-610) (which is indicated by analysis of mass spectrometry) with expanded structure due to intramolecular disulfide bond formation and glutathionylation. *D*, comparison of ^1^H-^15^N HSQC spectra for untreated control (-C, *black*), glutathionylated (-G, *red*), disulfide-bonded (-S-S-, *dark green*), and DTT-reduced (-D, *blue*) SBDα(537-610). CD, circular dichroism; HSQC, heteronuclear single quantum coherence; SEC, size-exclusion chromatography.

**Figure 8:**
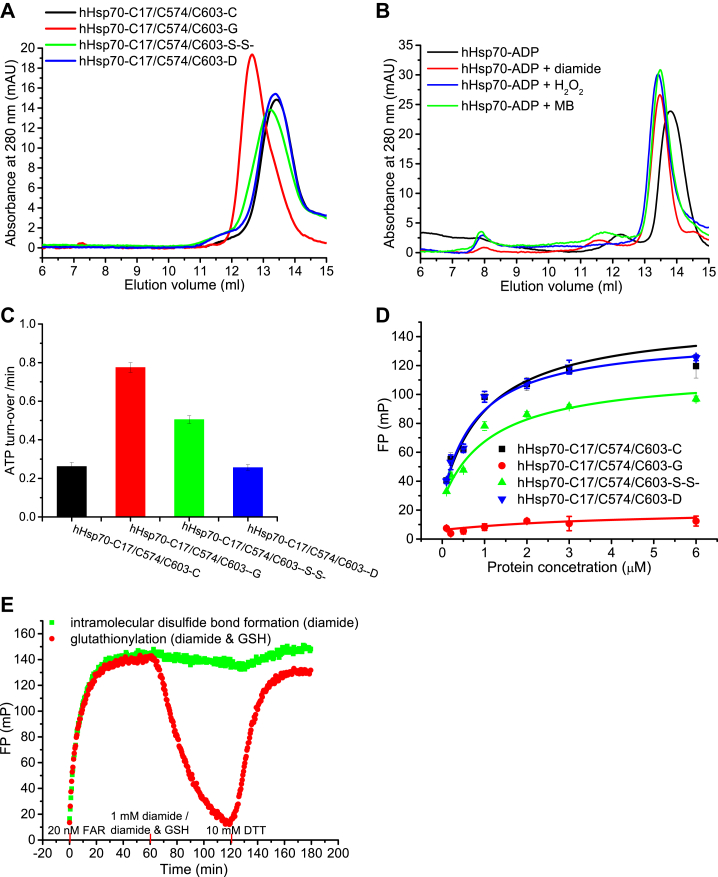
**Comparison of the effects of glutathionylation and intramolecular disulfide bond formation involving Cys574 and Cys603 on the structure and function of full length hHsp70.***A*, the effect of glutathionylation and intramolecular disulfide bond formation on conformation of hHsp70-C17/C574/C603 were compared by SEC analysis of untreated control (-C, *black*), glutathionylated (-G, *red*), disulfide-bonded (-S-S-, *green*), and DTT-reduced (-D, *blue*) hHsp70-C17/C574/C603. *B*, the effect of 2 mM H_2_O_2_, 1 mM methylene blue (MB), and 1 mM diamide on intramolecular disulfide bond formation of hHsp70 in the presence of 1 mM ADP were analyzed by SEC. In SEC analysis, the protein concentration was 10 μM. In SEC analysis, proteins of 10 μM were loaded onto a 24-ml Superdex 200 10/300 Gl column. The elution profiles were calibrated using blue dextran (2000 kDa, 7.30 ml), beta-amylase (200 kDa, 11.55 ml), alcohol dehydrogenase (150 kDa, 12.35 ml), bovine serum albumin (66 kDa, 13.54 ml), ovalbumin (45 kDa, 14.52 ml), carbonic anhydrase (29 kDa, 15.82 ml), and cytochrome c (12.4 kDa, 17.24 ml). The peak position of the hHsp70 monomer was 13.41 ml, and the peaks at 13.23 ml and 12.65 ml correspond to monomeric hHsp70 with expanded structure due to intramolecular disulfide bond formation and glutathionylation. *C*, the effect of glutathionylation and intramolecular disulfide bond formation on ATPase activity of hHsp70-C17/C574/C603 were compared by malachite green assay. *D*, the effect of glutathionylation and intramolecular disulfide bond formation on peptide binding of hHsp70-C17/C574/C603 were compared by fluorescence polarization (FP) assay. The data shown are the mean of three individual experiments and the error bars represent SEM. *E*, the effect of intramolecular disulfide bond formation (*green*) and glutathionylation (*red*) on kinetics of FAR peptide binding to ADP-bound hHsp70 were compared by FP assay. FAR peptide of 20 nM, 1 mM diamide, or 1 mM diamide with 1 mM GSH and 10 mM DTT were added sequentially at 0 min, 60 min, and 120 min time points to initiate peptide binding, intramolecular disulfide bond formation, or glutathionylation and then reduction, individually. SEC, size-exclusion chromatography.

Cys574 and Cys603 in the SBDα of hHsp70 can undergo multiple Cys modifications, including formation of an intramolecular disulfide bond and glutathionylation (21). Excess diamide and GSH facilitates disulfide bond formation between the GSH molecule and Cys residues in hHsp70 thus resulting in glutathionylation, but in the absence of sufficient GSH, diamide will lead to intramolecular disulfide bond formation between adjacent thiols in hHsp70 (21). In this study, we observed that intramolecular disulfide bond formation between Cys574 and Cys603 in hHsp70 SBDα(537-610) caused different structural changes compared to glutathionylation (Fig. 7). Compared to the effects of glutathionylation, the circular dichroism (CD) spectrum after intramolecular disulfide bond formation showed a smaller decrease in α-helical content (Fig. 7*A*) and the fluorescence spectrum was consistent with at least partial unfolding of the C-terminal domain (Fig. 7*B*). The distribution of the NMR peaks in the disulfide bond form of hHsp70 SBDα(537-610) was similar to the glutathionylated sample (Fig. 7*D*), but the signals were significantly weaker, indicating the existence of conformational exchange on an intermediate timescale. Combined with the observation by size-exclusion chromatography (SEC) that the hydrodynamic volume of the disulfide-bonded form of the C-terminal domain was intermediate between the unmodified and glutathionylated forms (Fig. 7*C*), these results suggest that, unlike the glutathionylated form, the disulfide-bonded form is only partially unfolded. The disulfide-bonded form of full length hHsp70 also showed an intermediate degree of structural and functional changes (*i.e.*, slight peak shift by SEC, moderately elevated ATPase activity, and slightly altered peptide binding) when compared to the unmodified and glutathionylated forms (Fig. 8). H_2_O_2_ and the Hsp70 inhibitor methylene blue (MB) induced a similar peak shift in SEC of ADP-bound hHsp70 with diamide (Fig. 8*B*), suggesting that H_2_O_2_ and MB also can cause intramolecular disulfide bond formation between Cys574 and Cys603 in the absence of GSH. Thus, under oxidative stress conditions, Cys574 and Cys603 can readily undergo intramolecular disulfide bond formation causing slight changes in the chaperone activity of hHsp70.

To determine whether Cys574 and Cys603 in the SBDα of hHsc70 can form an intramolecular disulfide bond, we analyzed the products of diamide treated or diamide and GSH-treated hHsc70 SBDα(524-616) by SEC and Q-TOF MS and found that both of the products were a mixture of two different components. The MS-detected molecular weight of untreated hHsc70 SBDα(524-616) was 10,954 Da, consistent with the molecular weight of protein containing reduced Cys574 and Cys603 (Fig. S4). Diamide-treated hHsc70 SBDα(524-616) contained intramolecular disulfide-bonded hHsc70 SBDα(524-616) (10,952 Da) and an undetermined component (11,018 Da) (Fig. S5). Diamide and GSH-treated hHsc70 SBDα(524-616) contained intramolecular disulfide-bonded hHsc70 SBDα(524-616) (10,952 Da) and glutathionylated hHsc70 SBDα(524-616) (11,565 Da) (Fig. S6). Consistent with this, diamide and GSH-treated ADP-bound hHsc70 caused only a slight increase in ATPase activity compared to ADP-bound hHsp70 (Fig. S2, *A* and *B*).

## Discussion

We have shown previously that in the presence of ADP, hHsp70 has much higher cysteine reactivity than hHsc70, and hHsp70 has higher glutathionylation and PES-modification rates than hHsc70 (21, 26). In this study, we thoroughly investigated the mechanisms of the differences in cysteine reactivity between hHsp70 and hHsc70 and found that cysteine reactivity of individual Cys residues was obviously lower in hHsc70 than in hHsp70, which is probably due to the lower structural dynamics of hHsc70. Further, we found that interaction between the SBDα and SBDβ had a strong inhibition effect on the cysteine reactivity of Cys574 and Cys603 in hHsc70 but not in hHsp70. We found that Gly557 in hHsp70 contributes significantly to the higher structural dynamics and cysteine reactivity of hHsp70 SBDα. The differences in cysteine reactivity between hHsp70 and hHsc70 may contribute to their different functions and functional regulation, which could provide strategies for designing specific Hsp70 inhibitors. Taken together, the results indicate that cysteine reactivity of Hsp70 is determined by structural dynamics, allosteric conformational changes, and domain communication. It is possible that factors including cochaperones, mutations (disease or evolution related), small molecules (including drugs), and other interaction partners, which could alter the above aspects of Hsp70, could also regulate cysteine reactivity of Hsp70 in complex cellular environments.

Exploring the cysteine reactivity of Hsp70 contributes to understanding of the response of Hsp70 to redox and electrophiles *in vivo*. We have previously identified the electrophile PES as a covalent inhibitor of hHsp70 and hHsc70 (26). In this study, we mainly used DTNB oxidation to evaluate the oxidative reactivity of thiols in hHsp70 and hHsc70. Although oxidative reactivity and electrophilic reactivity of thiols are often correlated, they are not equivalent. The lower DTNB reactivity of hHsc70 is consistent with its lower glutathionylation reactivity and lower PES reactivity than hHsp70 (21, 26). hHsp70-hHsc70(α) has higher DTNB reactivity and PES reactivity than WT hHsc70 (26). However, ATP/ADP and peptide substrate have different effects on DTNB reactivity and PES reactivity of hHsp70 (21, 26), and in this study, ATP also had different effects on DTNB reactivity of hHsp70 and hHsc70, indicating that the effect of allostery of Hsp70 on different types of thiol reaction and cysteine reactivity in different Hsp70 homologs can be specific.

Consistent with the cysteine reactivity detected by the DTNB reaction described here, different oxidative modifications of Cys residues of hHsp70 and hHsc70 in cells has also been reported using proteomics techniques and summarized in databases (23, 31, 32). Glutathionylation of all the five Cys residues in hHsp70 and all the four Cys in hHsc70 have been identified under normal or oxidative stress conditions (21, 23, 31, 33). Upon S-nitrosoglutathione (GSNO) treatment or nitrogen oxide signaling, S-nitrosylation of Cys17, Cys306, Cys574, and Cys603 in hHsp70 and Cys17, Cys574, and Cys603 in hHsc70 have been detected (20, 23, 34, 35, 36). Cys17, Cys574, and Cys603 in hHsc70 can be modified by S-sulfenation in living cells treated with H_2_O_2_, and Cys17 in hHsc70 was modified by S-sulfenation (-SO_2_H) or S-sulfonation (-SO_3_H) in cell lysates treated with H_2_O_2_ as detected by quantitative proteomic studies (37, 38, 39). Mutational studies and modification modeling of Cys17 in hHsc70 indicates that oxidative modification of Cys17 disrupts hydrogen-bond networks and impairs ATPase activity (40). The results reported here suggest that modification of Cys17 may occur secondary to the modification of the other Cys residues. S-sulfenation of Cys603 in hHsc70 was also detected (41). MB was found to cause S-sulfenation of Cys306 and secondary S-sulfenation of Cys267 in hHsp70 to inhibit ATPase activity but not in hHsc70 since hHsc70 lacks Cys306 (29). Persulfidation (S-sulfhydration, -SSH) of Cys306 and Cys574 in hHsp70 and Cys574 and Cys603 in hHsc70 upon hydrogen sulfide (H_2_S) signaling have also been identified (42, 43).

In this study, we observed that two different types of cysteine modification can occur under the same oxidative conditions. In hHsp70 or hHsc70, Cys574 and Cys603 can undergo glutathionylation or intramolecular disulfide bond formation, and these two different cysteine modifications potentially have different regulatory effects on chaperone activity. This suggests that different types of modification of specific Cys residues could coexist and conversion between different cysteine modifications is also possible, forming a more complex redox regulation network *in vivo*. The crosstalk between cysteine modifications and other PTMs of Hsp70 is still to be explored, that is, cysteine modifications of Hsp70 could alter its allostery and conformation to affect secondary PTMs.

Covalent modifications of hHsp70 and hHsc70 by electrophiles are also frequently identified, for example, 4-hydroxy-2-nonenal (4-HNE) can modify Cys267 of hHsp70 and inhibit the ATP affinity, substrate affinity, and luciferase refolding activity (44). Similarly, 2,5′-thiodipyrimidine and 5-(phenylthio) pyrimidine acrylamide derivatives were found to modify Cys267 to cause irreversible inhibition of hHsp70 (45). Covalent modification of Cys306 by natural small molecule handelin significantly elevates Hsp70 activity to enhance anti-neuroinflammation effects (46). As an α,β-unsaturated sesquiterpene lactone, parthenolide can cause covalent modification of Cys267 and Cys306 in hHsp70 (47). Cys574 and Cys603 in hHsp70 are also covalently targeted by PES and necroptosis-blocking 1 (NBC1) (26, 48). Thus, understanding the cysteine reactivity of hHsp70 and hHsc70 is important in order to predict the redox-regulated activity of chaperones *in vivo* and to design specific covalent inhibitors.

## Experimental procedures

### Protein expression and purification

The human *HSPA1A* gene (49) (UniProtKB code: P0DMV8) and *HSPA8* gene (UniProtKB code: P11142), which were kindly provided by Prof. Richard Morimoto, Northwestern University, were subcloned into the pET28a-smt3 expression plasmid for expression of hHsp70 and hHsc70 with a His6-Smt3 tag (50). All of the hHsp70 and hHsc70 mutants mentioned in this study (Table 1) were derived from the *HSPA1A*-pET28a-smt3 and *HSPA8*-pET28a-smt3 plasmids and were produced using an In-Fusion cloning method using a commercial kit (NEB).

**Table 1:** Summary of human HspA1A (hHsp70) and HspA8 (hHsc70) mutants used in this study and their outcomes

Name of protein	Description	Outcomes
WT hHsp70	hHsp70 (HspA1A/B)	Cysteine reactivity of Cys17, Cys267, Cys306, Cys574, and Cys603 of hHsp70
hHsp70-C17	hHsp70 C267S/C306S/C574A/C603A	Cysteine reactivity of Cys17 of hHsp70
hHsp70-C267	hHsp70 C17S/C306S/C574A/C603A	Cysteine reactivity of Cys267 of hHsp70
hHsp70-C306	hHsp70 C17S/C267S/C574A/C603A	Cysteine reactivity of Cys306 of hHsp70
hHsp70-C574	hHsp70 C17S/C267S/C306S/C603A	Cysteine reactivity of Cys574 of hHsp70
hHsp70-C603	hHsp70 C17S/C267S/C306S/C574A	Cysteine reactivity of Cys603 of hHsp70
hHsp70-C17/C267	hHsp70 C306S/C574A/C603A	Cysteine reactivity of Cys17 and Cys267 of hHsp70
hHsp70-C17/C306	hHsp70 C267S/C574A/C603A	Cysteine reactivity of Cys17 and Cys306 of hHsp70
hHsp70-C17/C574	hHsp70 C267S/C306S/C603A	Cysteine reactivity of Cys17 and Cys574 of hHsp70
hHsp70-C17/C603	hHsp70 C267S/C306S/C574A	Cysteine reactivity of Cys17 and Cys603 of hHsp70
hHsp70-C17/C574/C603	hHsp70 C267S/C306S	Cysteine reactivity of Cys17, Cys574, and Cys603 of hHsp70
hHsp70 SBDα(537-610)	hHsp70 Δ1-536/Δ611-641, SBDα of hHsp70 consisting of the remote part of α-helix B and α-helices C and D	Cysteine reactivity of Cys574 and Cys603 of hHsp70
hHsp70 SBDα(524-616)	hHsp70 Δ1-523/Δ617-641, SBDα of hHsp70 consisting of α-helix B, C, and D	Cysteine reactivity of Cys574 and Cys603 of hHsp70
hHsp70 SBDα(524-616)-C574	hHsp70 Δ1-523/Δ617-641/C603A	Cysteine reactivity of Cys574 of hHsp70
hHsp70 SBDα(524-616)-C603	hHsp70 Δ1-523/Δ617-641/C574A	Cysteine reactivity of Cys603 of hHsp70
hHsp70 SBDα(524-616) S551A/A552T/G557K/K559Q	hHsp70 Δ1-523/Δ617-641/S551A/A552T/G557K/K559Q	Cysteine reactivity of Cys574 and Cys603 of hHsp70
hHsp70 S551A/A552T/G557K/K559Q	hHsp70 S551A/A552T/G557K/K559Q	Cysteine reactivity of Cys17, Cys267, Cys306, Cys574, and Cys603 of hHsp70
hHsp70 SBDα(511-641)	hHsp70 Δ1-510, including the intact SBDα and C terminal random coil region of hHsp70	Cysteine reactivity of Cys574 and Cys603 of hHsp70
hHsp70 SBD(385-641)	hHsp70 Δ1-384, SBD of hHsp70	Cysteine reactivity of Cys574 and Cys603 of hHsp70
hHsp70(β)-hHsc70(α)	Chimera containing hHsp70 Δ1-384/Δ511-641 and hHsc70 Δ1-510	Cysteine reactivity of Cys574 and Cys603 of hHsc70
hHsp70-hHsc70(α)	Chimera containing hHsp70 Δ511-641 and hHsc70 Δ1-510	Cysteine reactivity of Cys17, Cys267, and Cys306 of hHsp70 and Cys574 and Cys603 of hHsc70
WT hHsc70	hHsc70 (HspA8)	Cysteine reactivity of Cys17, Cys267, Cys574, and Cys603 of hHsc70
hHsc70-no C	hHsc70 C17A/C267A/C574A/C603A	No cysteine reactivity
hHsc70-C17	hHsc70 C267A/C574A/C603A	Cysteine reactivity of Cys17 of hHsc70
hHsc70-C17/C267	hHsc70 C574A/C603A	Cysteine reactivity of Cys17 and Cys267 of hHsc70
hHsc70-C267	hHsc70 C17A/C574A/C603A	Cysteine reactivity of Cys267 of hHsc70
hHsc70-C574	hHsc70 C17A/C267A/C603A	Cysteine reactivity of Cys574 of hHsc70
hHsc70-C603	hHsc70 C17A/C267A/C574A	Cysteine reactivity of Cys603 of hHsc70
hHsc70-C574/C603	hHsc70 C17A/C267A	Cysteine reactivity of Cys574 and Cys603 of hHsc70
hHsc70 SBDα(524-616)	hHsc70 Δ1-523/Δ617-641, SBDα of hHsc70 consisting of α-helix B, C, and D	Cysteine reactivity of Cys574 and Cys603 of hHsc70
hHsc70 SBDα(524-616)-C574	hHsc70 Δ1-523/Δ617-641/C603A	Cysteine reactivity of Cys574 of hHsc70
hHsc70 SBDα(524-616)-C603	hHsc70 Δ1-523/Δ617-641/C574A	Cysteine reactivity of Cys603 of hHsc70
hHsc70 SBDα(524-616) A551S/T552A/K557G/Q559K	hHsc70 Δ1-523/Δ617-641/A551S/T552A/K557G/Q559K	Cysteine reactivity of Cys574 and Cys603 of hHsc70
hHsc70 A551S/T552A/K557G/Q559K	hHsc70 A551S/T552A/K557G/Q559K	Cysteine reactivity of Cys17, Cys267, Cys574, and Cys603 of hHsc70
hHsc70 SBDα(524-616) K557G	hHsc70 Δ1-523/Δ617-641/K557G	Cysteine reactivity of Cys574 and Cys603 of hHsc70
hHsc70 K557G	hHsc70 K557G	Cysteine reactivity of Cys17, Cys267, Cys574, and Cys603 of hHsc70
hHsc70 SBDα(511-646)	hHsc70 Δ1-510, including the intact SBDα and C terminal random coil region of hHsc70	Cysteine reactivity of Cys574 and Cys603 of hHsc70
hHsc70 SBD(385-646)	hHsc70 Δ1-384, SBD of hHsc70	Cysteine reactivity of Cys574 and Cys603 of hHsc70
hHsc70(β)-hHsp70(α)	Chimera containing hHsc70 Δ1-384/Δ511-646 and hHsp70 Δ1-510	Cysteine reactivity of Cys574 and Cys603 of hHsp70
hHsc70-hHsp70(α)	Chimera containing hHsc70 Δ511-646 and hHsp70 Δ1-510	Cysteine reactivity of Cys17 and Cys267 of hHsc70 and Cys574 and Cys603 of hHsp70

Expression and purification of hHsp70, hHsc70, and their mutants was performed as described (21, 25). Briefly, the proteins were purified twice by Ni-NTA column affinity purification before and after cleavage of His6-Smt3 by Ulp1 and then by a final SEC purification step. To make sure the cysteines in the purified proteins remain reduced after the purification steps, β-mercaptoethanol was added to the cell lysis buffer and to all of the buffers for Ni-NTA column purification to a final concentration of 2 mM. Further, DTT was added into the products of the second Ni-NTA column purification to a final concentration of 1 mM. To avoid the additional effect of DTT or β-mercaptoethanol on the Ellman assay, we used SEC running buffer B (50 mM Tris–HCl buffer, pH 7.5, containing 100 mM KCl and 5 mM MgCl_2_) which does not contain any reducing agents and so facilitates removal of the reducing agents included in the previous purification steps. All protein concentrations are given in terms of monomer and were determined using a bicinchoninic acid assay kit (Pierce).

### Measurement of cysteine reactivity (Ellman assay)

Cysteine reactivity of hHsp70, hHsc70, and their mutants was measured by Ellman assay as described. A standard curve was made using 0 to 100 μM free cysteine. DTNB (5 μl of 10 mM in 50 mM Na_2_HPO_4_/NaH_2_PO_4_ buffer, pH 7.5) was mixed with protein samples (145 μl of 10–20 μM) and the absorbance at 412 nm was measured in a SpectraMax M3e plate reader (Molecular Devices) or a Fluostar microplate reader (BMG Labtech) at room temperature (RT). If ADP or ATP was added, 1 mM ADP/ATP was mixed with WT or mutant hHsp70 or hHsc70 to give a total volume of 145 μl, and the mixture was allowed to stand at RT for 1 h before DTNB addition. For measurement of cysteine reactivity under denaturing conditions, 100 μl of 9 M urea in buffer B was mixed with the proteins and other components in a 50 μl volume to give a final urea concentration of 6 M. The number of active Cys residues can be calculated by dividing by the concentration of protein.

### Preparation of cysteine-modified Hsp70

To prepare glutathionylated and deglutathionylated Hsp70, 15 μM of WT or mutant hHsp70 or hHsc70 was mixed with 1 mM GSH and 1 mM diamide and allowed to stand in the dark at RT for 1 h in order to allow glutathionylation. Then, 10 mM DTT was added in order to deglutathionylate the protein. GSH, diamide, and DTT were then removed by dialysis. For glutathionylation of ADP-bound full length hHsp70, ADP (final concentration 1 mM) was added to the protein before GSH and diamide were added.

To prepare the disulfide-bonded form of Hsp70, 15 μM of WT or mutant hHsp70 or hHsc70 was mixed with 1 mM diamide, 2 mM H_2_O_2_, and 1 mM MB and allowed to stand in the dark at RT for 1 h in order to allow disulfide-bond formation. Then, 10 mM DTT was added in order to reduce the protein. Diamide and DTT were then removed by dialysis. For WT hHsp70, ADP (final concentration of 1 mM) was added to the protein before diamide was added.

Western blots were performed as standard to confirm glutathionylation and deglutathionylation of WT or mutant hHsp70 or hHsc70. Polyclonal anti-GSH (Millipore, AB5010) at 1:500 to 1000 dilution was used as the primary antibody for Western blot detection. Glutathionylation was also confirmed by the absence of free thiols by staining with maleimide functionalized Alexa Fluor 350 dye (AF 350 dye, blue fluorescence). The protein samples of 5 μl volume were mixed with 5 μl of buffer C (1 M Tris–HCl buffer, pH 7.5, containing 8% SDS (w/v) and 40% glycerin (v/v)) and then boiled for 10 min to destroy secondary structure. Cooled protein was mixed with 5 μl of the 200 μM AF 350 dye and incubated in the dark at RT for at least 2 h. A 5 μl volume of buffer D (1 M Tris–HCl buffer, pH 7.5, containing 0.4% Bromophenol Blue (w/v) and 40% glycerin (v/v)) was added and the mixture was boiled for another 5 min. Then, SDS-PAGE was performed to separate protein and surplus dye. Fluorescence of AF 350 dye was observed using excitation at 254 nm with a UV lamp.

MALDI-TOF MS was performed to detect glutathionylation of hHsp70-C306 and hHsp70-C17/C306. Glutathionylated peptide peaks can be distinguished in MALDI-TOF spectra by the corresponding 305-Da increase in molecular weight, when the samples are prepared without reducing agents such as DTT. Q-TOF MS was also performed to detect cysteine modifications of hHsc70 SBDα(524-616). Control, diamide- or diamide-and-GSH-treated, and DTT-reduced hHsc70 SBDα(524-616) (18 μl) was loaded onto the Q-TOF MS instrument after separation by HPLC. Profile spectra of 600-1800 M/Z were collected and deconvoluted using the software provided with the MS instrument (Thermo Scientific). The deconvoluting algorithm used was Maximum Entropy and the scale of molecular weight was 5000-80000 Da.

### Intrinsic fluorescence

Intrinsic fluorescence measurements were carried out on a Hitachi F-4500 or a Shimadzu RF-5301PC instrument. The intrinsic florescence spectra of glutathionylated, disulfide-bonded, and reduced hHsp70 or its mutants were measured between 310 and 380 nm using excitation wavelengths of 295 nm or measured between 290 and 400 nm using excitation wavelengths of 280 nm at 25 °C. The proteins were prepared in buffer B.

### Circular dichroism

Far-UV CD spectra were measured between 200 and 250 nm on a Chirascan Plus CD instrument (Applied Photophysics) at 25 °C in a 1 mm path-length thermostatted cuvette after preincubation for 10 min at 25 °C. Spectra of glutathionylated, disulfide-bonded, and reduced hHsp70 or its mutants were compared in buffer B.

### Size-exclusion chromatography

The oligomeric state of glutathionylated, disulfide-bonded, and reduced WT or mutant hHsp70 or hHsc70 were compared by SEC using a Superdex 200 10/300 Gl 24 ml column (GE) for full length Hsp70 proteins or a Superdex 75 10/300 Gl 24 ml column (GE) for truncated Hsp70 mutants, in buffer B at RT. Blue dextran (2000 kDa), beta-amylase (200 kDa), alcohol dehydrogenase (150 kDa), bovine serum albumin (66 kDa), ovalbumin (45 kDa), carbonic anhydrase (29 kDa), PMSF-treated trypsinogen (24 kDa), and cytochrome c (12.4 kDa) were used as molecular mass standards.

### NMR experiments

^15^N/^13^C-labeled hHsp70 SBDα(524-616) and hHsc70 SBDα(524-616) were prepared using a similar protocol as described (21). NMR samples of hHsp70 SBDα(524-616) and hHsc70 SBDα(524-616) contained 0.6 mM protein in buffer A (25 mM Na_2_HPO_4_-NaH_2_PO_4_, 50 mM NaCl, pH 7.0), with addition of 2 mM EDTA, 5 mM DTT, and 0.02% (w/v) sodium 2,2-dimethylsilapentane-5-sulfonate in 10% (v/v) D_2_O. NMR experiments were performed at RT on an Agilent DD2 600 MHz spectrometer equipped with cryo-probe. Backbone assignments of hHsp70 SBDα(524-616) and hHsc70 SBDα(524-616) were obtained from experiments including the two-dimensional ^1^H-^15^N and ^1^H-^13^C heteronuclear single quantum coherence, and the three-dimensional CBCA(CO)NH, HNCACB, HNCO, and HN(CA)CO. The longitudinal relaxation rates (R1), transverse relaxation rates (R2), and steady-state heteronuclear ^1^H-^15^N NOE values of hHsp70 SBDα(524-616) and hHsc70 SBDα(524-616) were measured using standard pulse programs (51). The delays used for the R1 experiments were 10, 100, 300, 500, 800, 1000, 1200, 1600, and 2000 ms, and those used for the R2 experiments were 7.2, 21.6, 36, 50.4, 64.8, 79.2, 93.6, and 108 ms. The relaxation rate constants were obtained by fitting the peak intensities to a single exponential function using the RateAnalysis module in NMRViewJ (52).

### ATPase assay (malachite green)

Colorimetric determination of inorganic phosphate produced by ATP hydrolysis was performed using the malachite green reagent, prepared as described (53, 54). A 10-μl volume of glutathionylated, disulfide-bonded, or reduced WT or mutant hHsp70 or hHsc70 (1 μM) was mixed with 10 μl of 2 mM ATP in buffer B in a 96-well plate. The plate was incubated for 4 h at 37 °C. An 80-μl volume of malachite green solution and 10 μl of 34% sodium citrate were added sequentially. The samples were mixed thoroughly and incubated at 37 °C for 30 min before measuring the A_620_ on a SpectraMax M3e plate reader (Molecular Devices). The rate of intrinsic ATP hydrolysis was deduced by subtracting the signal from ATP in the absence of chaperone.

### ATP agarose-binding assay

Glutathionylated or deglutathionylated hHsp70 or its mutants (2 μM) in buffer B were loaded onto an ATP agarose column which was equilibrated with buffer B. Nonbinding protein was washed with buffer B and collected in the run-through. ADP (4 mM) in buffer B was applied to elute the bound protein. The run-through and elution fractions were checked by SDS-PAGE to evaluate the binding of glutathionylated or deglutathionylated hHsp70 or its mutants to ATP/ADP.

### Peptide-binding assay

Peptide-binding assays based on fluorescence polarization (FP) were performed as described previously (21, 55). Steady-state FP measurements were performed at RT with 60-min incubation in buffer B to give the dissociation constant (*K*_D_). Binding was assessed by incubating the increasing concentrations of control, glutathionylated, or deglutathionylated hHsp70 or its mutants with a fixed concentration (20 nM) of fluorescently-labeled substrate (FITC-ALLLSAPRR peptide, FAR) and FP values were measured. FP measurements were performed on a Fluostar microplate reader (BMG Labtech) using the FP filter set (emission 485 and excitation 520 nm). FP values are expressed in millipolarization (mP) units. All statistical analyses were performed with Origin 9 software (https://www.originlab.com/). Binding data were analyzed using nonlinear regression analysis (single site–binding model) in Origin 9. Kinetic FP measurements were performed by monitoring the time course of peptide binding at RT. After rapid mixing of 20 nM FAR and 10 μM hHsp70 or its mutants in the absence or in the presence of 1 mM ADP or ATP, FP was recorded against time. Peptide-bound hHsp70 (or its mutants) in the absence or presence of nucleotide was glutathionylated (or oxidized) by addition of 1 mM diamide with 1 mM GSH (or 1 mM diamide alone) at the 60 min time point and then deglutathionylated or reduced by addition of 10 mM DTT at the 120 min time point.

## Data availability

All data are contained within the article.

## Supporting information

This article contains supporting information.

## Conflict of interest

The authors declare that they have no conflicts of interest with the contents of this article.
