# Supplementary material for: Exploration of the cysteine reactivity of human inducible Hsp70 and cognate Hsc70
Source: J Biol Chem. 2022 Nov 19;299(1):102723. doi: 10.1016/j.jbc.2022.102723 (PMC9800336; doi:10.1016/j.jbc.2022.102723)
Supplement: Supporting information [file mmc1.docx]

**Supporting Information for:**

**Exploration of the cysteine reactivity of human inducible Hsp70 and cognate Hsc70**

Zhouping Hong (洪舟萍)^1#^**^§^**, Weibin Gong (宫维斌)^1^**^§^**, Jie Yang (杨杰)^1,2#^, Sainan Li (李赛楠)^1,2#^, Zhenyan Liu (刘珍艳)^1^, Sarah Perrett (柯莎)^1,2^* and Hong Zhang (张红)^1,2^*

^1^National Laboratory of Biomacromolecules, CAS Center for Excellence in Biomacromolecules, Institute of Biophysics, Chinese Academy of Sciences, 15 Datun Road, Chaoyang District, Beijing 100101, China.

^2^University of the Chinese Academy of Sciences, 19 Yuquan Road, Shijingshan District, Beijing 100049, China.

^#^Present addresses: Z. Hong, Department of Cell Biology, Yale School of Medicine, New Haven, CT 06520, USA.; J. Yang, Leewe Biopharmaceutical Co., Ltd., Xianlin University Town, Jiangsu Life Park 7th floor F2 building, 9 Weidi Road, Qixia District, Nanjing, 210033, China; S. Li, Key Laboratory of Biofuels and Shandong Provincial Key Laboratory of Synthetic Biology, Qingdao Institute of Bioenergy and Bioprocess Technology, Chinese Academy of Sciences, 189 Songling Road, Laoshan District, Qingdao, Shandong 266101, China; H. Zhang, State Key Laboratory of Medical Molecular Biology, Institute of Basic Medical Sciences, Chinese Academy of Medical Sciences, School of Basic Medicine, Peking Union Medical College, Beijing 100005, China.

*To whom correspondence should be addressed at: National Laboratory of Biomacromolecules, Institute of Biophysics, Chinese Academy of Sciences, 15 Datun Road, Chaoyang District, Beijing 100101, China. Tel.: +86-10-64888496; Email addresses: zhangh@moon.ibp.ac.cn, sarah.perrett@cantab.net

**^§^**These authors contributed equally to this work.

A

B

C

D

E

F

G

H

I

J

K

L

**Figure S1. The effect of glutathionylation on the conformation of hHsp70.** Untreated control (-C, black), glutathionylated (-G, red) and deglutathionylated (-D, blue) WT hHsp70 or its mutants, as indicated, were examined by SEC (**A**-**C, G-I**) or intrinsic fluorescence (**D-E, J-L**). In SEC analysis, proteins of 10 μM concentration were loaded onto a 24-ml Superdex 200 10/300 GL column. The elution profiles were calibrated using the elution volumes of blue dextran (2000 kDa, 7.30 ml), beta-amylase (200 kDa, 11.55 ml), alcohol dehydrogenase (150 kDa, 12.35 ml), bovine serum albumin (66 kDa, 13.54 ml), ovalbumin (45 kDa, 14.52 ml) carbonic anhydrase (29 kDa, 15.82 ml) and cytochrome c (12.4 kDa, 17.24 ml). The peak position of the hHsp70 monomer was 13.41 ml, and the peak at 11.66 ml corresponds to a mixture of dimeric and trimeric hHsp70. The elution peak around 7-8 ml is large oligomers of hHsp70 induced by glutathionylation. The peak at around 12.80 ml corresponds to the monomeric hHsp70 with expanded structure due to glutathionylation. In intrinsic fluorescence measurements the protein concentration was 2 μM, and the excitation wavelength was 280 nm.

A

B

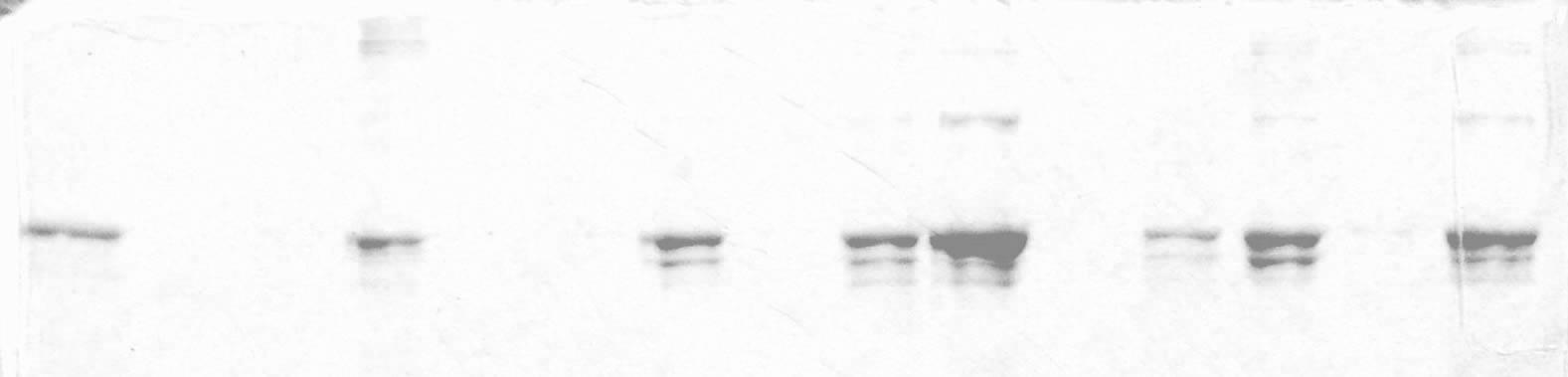

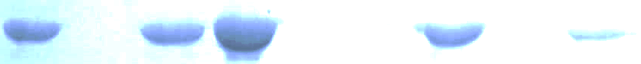


hHsp70-C

FT W E FT W E FT W E FT W E FT W E FT W E

hHsp70-G

hHsp70-D

C17/C306-C

C17/C306-G

C17/C306-D


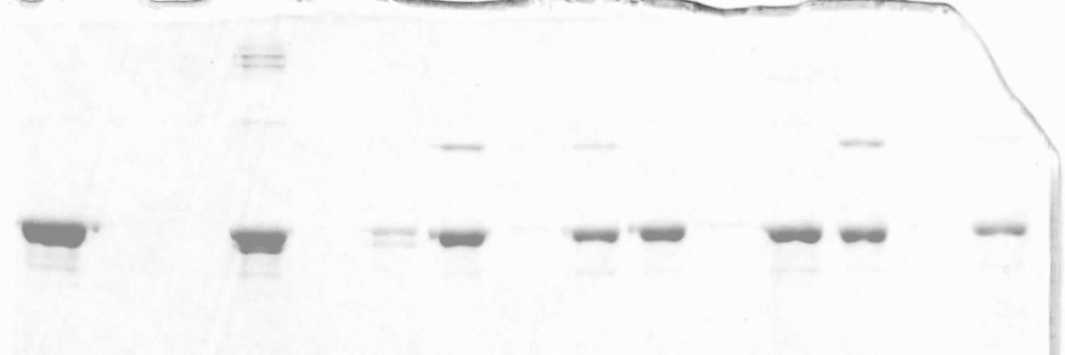

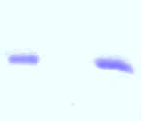


FT W E FT W E FT W E FT W E FT W E FT W E

C17/C267-C

C17/C267-G

C17/C267-D

C17/C574/C603-C

C17/C574/C603-G

C17/C574/C603-D

D

E

G

FD

I

H

C

**Figure S2. The effect of glutathionylation on function of hHsp70 and hHsc70.** (**A**) The effect of glutathionylation of individual and combinations of Cys17, Cys267, Cys306, Cys574 and Cys603 on ATPase activity of hHsp70 were detected. ATPase activity of untreated control (-C, black), glutathionylated (-G, red) and deglutathionylated (-D, blue) WT hHsp70 and hHsp70 mutants were measured by malachite green assay. (**B**) The effect of glutathionylation on ATPase activity of hHsc70 were detected by malachite green assay. The glutathionylation treatment was performed in the absence of nucleotide or in the presence of 1 mM ADP as indicated. (**C**) The effect of glutathionylation of individual Cys residues on the nucleotide binding ability of hHsp70 was detected by ATP agarose binding assay. Binding to ATP agarose and elution by ADP of untreated control (-C), glutathionylated (-G) and deglutathionylated (-D) WT hHsp70, hHsp70-C17/C267, hHsp70-C17/C306, hHsp70-C17/C574/C603 was checked by SDS-PAGE. RT, run through; W, wash; E, eluate. (**D-I**) The effect of glutathionylation of individual Cys residues on peptide binding ability of hHsp70 was detected by fluorescence polarization (FP) assay. The data shown are the mean of three individual experiments and the error bars represent the standard error of the mean.

A

B

C

D

E

F

G

H

I

J

K

**Figure S3.** The effect of glutathionylation on the conformation of hHsc70. (**A-K**) Untreated control (-C, black), glutathionylated (-G, red) and deglutathionylated (-D, blue) WT hHsc70 or its mutants, in the absence of nucleotide (**A-G**) or in the presence of 1 mM ADP (**H-K**) as indicated, were examined by SEC. The protein concentration was 10 μM. The peak position of the hHsc70 monomer was 13.21 ml, and the peaks of 11.50 ml correspond to a mixture of dimeric and trimeric hHsc70. The elution peak around 7-8 ml is large oligomers of hHsc70 induced by glutathionylation. The peaks at 12.28 ml and 10.80 ml correspond to monomeric and oligomeric hHsc70, respectively, with expanded structure due to glutathionylation. Other details are as described in the legend to Figure S1.

**Figure S4.** The molecular weight of the purified hHsc70 SBDα(524-616) without any treatment was analyzed by Q-TOF mass spectrometry.

A

B

C

**Figure S5.** Detection of disulfide bond formation between Cys574 and Cys603 in hHsc70. (**A-B**) After treatment with diamide (**A**) and additional treatment with DTT (**B**), the molecular weight of the hHsc70 SBDα(524-616) was analyzed by Q-TOF mass spectrometry. (**C**) Conformation of untreated control (-C, black), diamide treated (-diamide, red), and reduced after diamide treatment (-diamide-DTT, blue) hHsc70 SBDα(524-616) were compared by SEC. Proteins of 20 μM were loaded onto a 24-ml Superdex 75 10/300 GL column. The elution profiles were calibrated using blue dextran (2000 kDa, 7.50 ml), beta-amylase (200 kDa, 8.35 ml), alcohol dehydrogenase (150 kDa, 8.80 ml), bovine serum albumin (66 kDa, 9.56 ml), ovalbumin (45 kDa, 10.48 ml) carbonic anhydrase (29 kDa, 11.79 ml), PMSF-treated trypsinogen (24 kDa, 12.60 ml) and cytochrome c (12.4 kDa, 13.54 ml). The peak position of monomeric hHsc70 SBDα(524-616) (11 kDa) was 13.67 ml, and the peak at 12.80 ml corresponds to monomeric hHsc70 SBDα(524-616) (confirmed by mass spectrometry) with expanded structure due to cysteine modifications.

A

B


C

**Figure S6.** Detection of glutathionylation of Cys574 and Cys603 in hHsc70. (**A-B**) After treatment with diamide and GSH (**A**) and additional treatment with DTT (**B**), the molecular weight of the hHsc70 SBDα(524-616) was analyzed by Q-TOF mass spectrometry. (**C**) Conformation of untreated control (-C, black), diamide and GSH treated (-diamide + GSH, red), and reduced after diamide and GSH treatment (-diamide + GSH -DTT, blue) hHsc70 SBDα(524-616) were compared by SEC. The protein concentration was 20 μM. The peak position of the hHsc70 SBDα(524-616) monomer (11 kDa) was 13.67 ml, and the peak at 11.88 ml corresponds to monomeric hHsc70 SBDα(524-616) (confirmed by mass spectra) with expanded structure due to cysteine modifications. Other details are as described in the legend to Figure S5.

**Table S1. FAR peptide binding constants for hHsp70 and its mutants**

|  | | ***K*_D_ (μM)** |
| --- | --- | --- |
| WT | Control | 0.96±0.12 |
|  | Glutathionylated | ND^a^ |
|  | DTT reduced | 1.94±0.14 |
| hHsp70-C17/C267 | Control | 1.35±0.23 |
|  | Glutathionylated | ND^a^ |
|  | DTT reduced | 3.18±0.55 |
| hHsp70- C17/C306 | Control | 1.12±0.15 |
|  | Glutathionylated | 1.71±0.41 |
|  | DTT reduced | 1.36±0.43 |
| hHsp70- C17/C574 | Control | 1.25±0.08 |
|  | Glutathionylated | ND^a^ |
|  | DTT reduced | 1.34±0.06 |
| hHsp70- C17/C603 | Control | 1.06±0.09 |
|  | Glutathionylated | ND^a^ |
|  | DTT reduced | 1.13±0.14 |
| hHsp70- C17/C574/C603 | Control | 1.35±0.23 |
|  | Glutathionylated | ND^a^ |
|  | -S-S- | 1.15±0.44 |
|  | DTT reduced | 1.47±0.21 |

^a^ ND, not determined.
